# Supplementary material for: Cortical dynein pulling mechanism is regulated by differentially targeted attachment molecule Num1
Source: eLife. 2018 Aug 7;7:e36745. doi: 10.7554/eLife.36745 (PMC6080947; doi:10.7554/eLife.36745)
Supplement: Supplementary file 1. [file elife-36745-supp1.docx]

**Supplementary file 1.** Yeast strains used in this study

| Strain | Genotype | Source |
| --- | --- | --- |
| YWL36 | *MATa ura3-52 lys2-801 leu2-∆1 his3-∆200 trp1-∆63* | Vorvis et al., 2008 |
| YWL37 | *MATα ura3-52 lys2-801 leu2-∆1 his3-∆200 trp1-∆63* | Vorvis et al., 2008 |
| YWL630 | *MATa NUM1-yEGFP::spHIS5 ura3-52 lys2-801 leu2-∆1 his3-∆200 trp1-∆63* | Tang et al., 2009 |
| YWL2228 | *MATa MET3p:mCherry-TUB1::URA3 DYN1-3GFP::TRP1 ura3-52 lys2-801 leu2-∆1 his3-∆200 trp1-∆63* | Tang et al., 2009 |
| YWL10 | *MATα DYN1-linker-TAP::TRP1 ura3-52 trp1 lys2-801 leu2∆1 his3∆200 pep4::HIS3 prb1∆1.6R can1 GAL* | This study |
| YWL521 | *MATα dyn1∆::HIS3 ura3-52 lys2-801 leu2-∆1 his3-∆200 trp1-∆63* | This study |
| YWL2572 | *MATα NUM1-yEGFP::spHIS5 COX4-mCherry::URA3 ura3-52 lys2-801 leu2-∆1 his3-∆200 trp1-∆63* | This study |
| YWL3090 | *MATα JNM1-13myc::HPH^R^ ura3-52 trp1 lys2-801 leu2∆1 his3∆200 pep4::HIS3 prb1∆1.6R can1 GAL* | This study |
| YWL3792 | *MATα num1^L167E+L170E^::LEU2 MET3p:mCherry-TUB1::URA3 DYN1-3GFP::TRP1 ura3-52 lys2-801 leu2-∆1 his3-∆200 trp1-∆63* | This study |
| YWL4606 | *MATa TEF1p:BIP-EGFP-HDEL::URA3 lys2-801 leu2-∆1 his3-∆200 trp1-∆63* | This study |
| YWL4612 | *MATα num1∆::spHIS5 TEF1p:BIP-EGFP-HDEL::URA3 lys2-801 leu2-∆1 his3-∆200 trp1-∆63* | This study |
| YWL4759 | *MATa mCherry-TUB1::LEU2 ura3-52 lys2-801 leu2-∆1 his3-∆200 trp1-∆63* | This study |
| YWL4760 | *MATα num1∆::HIS3 mCherry-TUB1::LEU2 ura3-52 lys2-801 leu2-∆1 his3-∆200 trp1-∆63* | This study |
| YWL4764 | *MATa NUM1-yEGFP::spHIS5 scs2∆::TRP1 scs22∆::KAN^R^ ura3-52 lys2-801 leu2-∆1 his3-∆200 trp1-∆63* | This study |
| YWL4769 | *MATα* *dyn1∆::TRP1 HIS3p:mRuby2-TUB1+3'UTR::HPH^R^ ura3-52 lys2-801 leu2-∆1 his3-∆200 trp1-∆63* | This study |
| YWL4774 | *MATa NUM1-yEGFP::spHIS5 HIS3p:mRuby2-TUB1+3'UTR::HPH^R^ ura3-52 lys2-801 leu2-∆1 his3-∆200 trp1-∆63* | This study |
| YWL4778 | *MATa NUM1-yEGFP::spHIS5 COX4-mCherry::URA3 scs22∆::KAN^R^ ura3-52 lys2-801 leu2-∆1 his3-∆200 trp1-∆63* | This study |
| YWL4779 | *MATa NUM1-yEGFP::spHIS5 COX4-mCherry::URA3 scs22∆::KAN^R^ scs2∆::TRP1 ura3-52 lys2-801 leu2-∆1 his3-∆200 trp1-∆63* | This study |
| YWL4783 | *MATa* *NUM1-yEGFP::spHIS5 scs2∆::TRP1 scs22∆::KAN^R^ HIS3p:Venus-TUB1+3'UTR::URA3 ura3-52 lys2-801 leu2-∆1 his3-∆200 trp1-∆63* | This study |
| YWL4865 | *MATa scs2∆::TRP1 scs22∆::HPH^R^ TEF1p:BIP-EGFP-HDEL::URA3 ura3-52 lys2-801 leu2-∆1 his3-∆200 trp1-∆63* | This study |
| YWL4885 | *MATα NUM1-13myc::HPH^R^ GFP-TUB1::LEU2 ura3-52 trp1 lys2-801 leu2∆1 his3∆200 pep4::HIS3 prb1∆1.6R can1 GAL* | This study |
| YWL4887 | *MATα NUM1-13myc::HPH^R^ scs2∆::TRP1 scs22∆::KAN^R^ GFP-TUB1::LEU2 ura3-52 trp1 lys2-801 leu2∆1 his3∆200 pep4::HIS3 prb1∆1.6R can1 GAL* | This study |
| YWL4888 | *MATα NUM1-yEGFP::HIS5 kar9∆::KAN^R^ scs2∆::TRP1 scs22∆::HPH^R^ TUB1-GFP ura3-52 lys2-801 leu2-∆1 his3-∆200 trp1-∆63* | This study |
| YWL4912 | *MATα scs2∆::TRP1 scs22∆::KAN^R^ dyn1∆::HIS5 ura3-52 lys2-801 leu2-∆1 his3-∆200 trp1-∆63* | This study |
| YWL4915 | *MATα DYN1-3GFP::TRP1 scs22∆::KAN^R^ scs2∆::TRP1 HIS3p:mRuby2-TUB1+3'UTR::LEU2 ura3-52 lys2-801 leu2-∆1 his3-∆200 trp1-∆63* | This study |
| YWL4921 | *MATα NUM1-yEGFP::HIS5 kar9∆::KAN^R^ scs2∆::TRP1 scs22∆::HPH^R^ HIS3p:mRuby2-TUB1+3'UTR::LEU2 ura3-52 lys2-801 leu2-∆1 his3-∆200 trp1-∆63* | This study |
| YWL4924 | *MATa num1^L167E+L170E^::LEU2 MET3:mCherry-TUB1::URA3 DYN1-3GFP::TRP1 scs2∆::TRP1 scs22∆::KAN^R^ ura3-52 lys2-801 leu2-∆1 his3-∆200 trp1-∆63* | This study |
| YWL4949 | *MATα kar9∆::HIS3 ura3-52 lys2-801 leu2-∆1 his3-∆200 trp1-∆63* | This study |
| YWL4957 | *MATα JNM1-13myc::HPH^R^ scs2∆::TRP1 scs22∆::KAN^R^ ura3-52 trp1 lys2-801 leu2∆1 his3∆200 pep4::HIS3 prb1∆1.6R can1 GAL* | This study |
| YWL4958 | *MATα num1^L167E+L170E^-yEGFP::spHIS5 scs2∆::TRP1 HIS3p:mRuby2-TUB1+3'UTR::URA3 scs22∆::KAN^R^ ura3-52 lys2-801 leu2-∆1 his3-∆200 trp1-∆63* | This study |
| YWL4959 | *MATα num1^L167E+L170E^-yEGFP::spHIS5 HIS3p:mRuby2-TUB1+3'UTR::URA3 ura3-52 lys2-801 leu2-∆1 his3-∆200 trp1-∆63* | This study |
| YWL4963 | *MATa DYN1-3GFP::TRP1 kar9∆::HIS3 scs22∆::KAN^R^ scs2∆::TRP1 HIS3p:mRuby2-TUB1+3'UTR::LEU2 ura3-52 lys2-801 leu2-∆1 his3-∆200 trp1-∆63* | This study |
| YWL4965 | *MATα NUM1-yEGFP::HIS5 kar9∆::KAN^R^ HIS3p:mRuby-TUB1+3'UTR::URA3 ura3-52 lys2-801 leu2-∆1 his3-∆200 trp1-∆63* | This study |
| YWL4966 | *MATα NUM1-yEGFP::HIS5 kar9∆::KAN^R^ HIS3p:mRuby-TUB1+3'UTR::URA3 ura3-52 lys2-801 leu2-∆1 his3-∆200 trp1-∆63* | This study |
| YWL4983 | *MATα scs2∆::TRP1 scs22∆::KAN^R^ dyn1∆::HIS5 HIS3p:mRuby2-TUB1+3'UTR::URA3 ura3-52 lys2-801 leu2-∆1 his3-∆200 trp1-∆63* | This study |
| YWL5033 | *MATα DYN1-3GFP::TRP1 scs2∆::TRP1 scs22∆::KAN^R^ pac1∆::URA3 HIS3p:mRuby2-TUB1+3'UTR::LEU2 ura3-52 lys2-801 leu2-∆1 his3-∆200 trp1-∆63* | This study |
| YWL5034 | *MATα DYN1-3GFP::TRP1 scs22∆::KAN^R^ scs2∆::TRP1 kip2∆::HIS5 HIS3p:mRuby2-TUB1+3'UTR::LEU2 ura3-52 lys2-801 leu2-∆1 his3-∆200 trp1-∆63* | This study |
| YWL5036 | *MATα DYN1-3GFP::TRP1 scs2∆::TRP1 scs22∆::KAN^R^ arp1∆::HIS5 HIS3p:mRuby2-TUB1+3'UTR::LEU2 ura3-52 lys2-801 leu2-∆1 his3-∆200 trp1-∆63* | This study |
| YWL5048 | *MATa kar9∆::TRP1 DYN1-3GFP::TRP1 HIS3p:mRuby2-TUB1+3'UTR::LEU2 ura3-52 lys2-801 leu2-∆1 his3-∆200 trp1-∆63* | This study |
| YWL5037 | *MATα DYN1-3GFP::TRP1 kar9∆::His3 kip3∆::HPH^R^ scs2∆::TRP1 scs22∆::KAN^R^ HIS3p:mRuby2-TUB1+3'UTR::LEU ura3-52 lys2-801 leu2-∆1 his3-∆200 trp1-∆63* | This study |
| YWL5084 | *MATa* *NUM1-yEGFP::spHIS5 ist2∆::HPH^R^ kar9∆::KAN^R^ HIS3p:mRuby2-TUB1+3'UTR::LEU2 ura3-52 lys2-801 leu2-∆1 his3-∆200 trp1-∆63* | This study |
| YWL5087 | *MATα NUM1-yEGFP-CAAX::spHIS5 scs22∆::KAN^R^ scs2∆::TRP1 HIS3p:mRuby2-TUB1+3'UTR::LEU2 kar9∆::HPH^R^ ura3-52 lys2-801 leu2-∆1 his3-∆200 trp1-∆63* | This study |
| YWL5116 | *MATα DYN1-linker-TAP::TRP1 scs2∆::KAN^R^ scs22∆::URA3 TUB1-GFP::LEU2 ura3-52 trp1 lys2-801 leu2∆1 his3∆200 pep4::HIS3 prb1∆1.6R can1 GAL* | This study |
| YWL5126 | *MATα NUM1-yEGFP::HIS5 kar9∆::KAN^R^ scs2∆::TRP1 scs22∆::HPH^R^ kar3∆::URA3 HIS3p:mRuby2-TUB1+3'UTR::LEU2 ura3-52 lys2-801 leu2-∆1 his3-∆200 trp1-∆63* | This study |
| YWL5226 | *MATa dyn1∆::HIS3 dyn1^K2424A^-3YFP::TRP1 scs2∆::TRP1 scs22∆::KAN^R^ HIS3p:mRuby2-TUB1+3'UTR::LEU2 ura3-52 lys2-801 leu2-∆1 his3-∆200 trp1-∆63* | This study |
| YWL5318 | *MATa NUM1-yEGFP-CAAX::spHIS5 kar9∆::KAN^R^ HIS3p:mRuby2-TUB1+3'UTR::LEU2 ura3-52 lys2-801 leu2-∆1 his3-∆200 trp1-∆63* | This study |
| YWL5361 | *MATa NUM1-yEGFP::spHIS5 scs2∆::TRP1 scs22∆::KAN^R^ HIS3p:mRuby2-TUB1+3'UTR::URA3 ura3-52 lys2-801 leu2-∆1 his3-∆200 trp1-∆63* | This study |
| YWL5439 | *MATa NUM1-yEGFP::spHIS5 nip100∆aa(2-103) kar9∆::HPH^R^ HIS3p:mRuby2-TUB1+3'UTR::URA3 ura3-52 lys2-801 leu2-∆1 his3-∆200 trp1-∆63* | This study |
| YWL5443 | *MATa NUM1-yEGFP::spHIS5 scs2∆::TRP1 scs22∆::KAN^R^ nip100∆aa(2-103) kar9∆::HPH^R^ HIS3p:mRuby2-TUB1+3'UTR::URA3 ura3-52 lys2-801 leu2-∆1 his3-∆200 trp1-∆63* | This study |
| YWL5488 | *MATα* *DYN1-3GFP::TRP1 nip100∆aa(2-103) scs2∆::KAN^R^ scs22∆::HPH^R^ HIS3p:mRuby2-TUB1+3'UTR::URA3 ura3-52 lys2-801 leu2-∆1 his3-∆200 trp1-∆63* | This study |
| YWL5542 | *MATa NUM1-yEGFP::spHIS5 COX4-mCherry::URA3 scs22∆::KAN^R^ scs2∆::TRP1 mmr1∆::HPH^R^ ura3-52 lys2-801 leu2-∆1 his3-∆200 trp1-∆63* | This study |
| YWL5555 | *MATa NUM1-yEGFP::spHIS5 COX4-mCherry::URA3 scs22∆::KAN^R^ scs2∆::TRP1 mmr1∆::HPH^R^ gem1∆::LEU2 ura3-52 lys2-801 leu2-∆1 his3-∆200 trp1-∆63* | This study |
| YWL5197 | *MATa NUM1-yEGFP::spHIS5 scs2∆::TRP1 bni1∆::KAN^R^ scs22∆::KAN^R^ HIS3p:mRuby2-TUB1+3'UTR::LEU2 ura3-52 lys2-801 leu2-∆1 his3-∆200 trp1-∆63* | This study |
| YWL5203 | *MATα NUM1-yEGFP::spHIS5 bni1∆::TRP1 HIS3p:mRuby2-TUB1+3'UTR::URA3 ura3-52 lys2-801 leu2-∆1 his3-∆200 trp1-∆63* | This study |
| YWL5361 | *MATa NUM1-yEGFP::spHIS5 scs2∆::TRP1 scs22∆::KAN^R^ HIS3p:mRuby2-TUB1+3'UTR::URA3 ura3-52 lys2-801 leu2-∆1 his3-∆200 trp1-∆63* | This study |
| YWL706 | *MATα NUM1-yEGFP::spHIS5 ura3-52 lys2-801 leu2-∆1 his3-∆200 trp1-∆63* | This study |
| YWL1673 | *MATα JNM1-3mCherry::HIS3 CFP-TUB1::URA3 ura3-52 lys2-801 leu2-∆1 his3-∆200 trp1-∆63* | This study |
| YWL5267 | *MATα JNM1-3mCherry::HIS3 scs2∆::TRP1 scs22∆::KAN^R^ CFP-TUB1::URA3 ura3-52 lys2-801 leu2-∆1 his3-∆200 trp1-∆63* | This study |
| YWL3955 | *MATa cin8∆::HIS3 GFP-TUB1::LEU2 ura3-52 lys2-801 leu2-∆1 his3-∆200 trp1-∆63* | This study |
| YWL4866 | *MATα scs2∆::TRP1 scs22∆::KAN^R^ ura3-52 lys2-801 leu2-∆1 his3-∆200 trp1-∆63* | This study |
| YWL4751 | *MATa NUM1-yEGFP::spHIS5 scs2∆::TRP1 ura3-52 lys2-801 leu2-∆1 his3-∆200 trp1-∆63* | This study |
| YWL5593 | *MATα NUM1-yEGFP::spHIS5 SCS2-mRuby2::KAN^R^ ura3-52 lys2-801 leu2-∆1 his3-∆200 trp1-∆63* | This study |
| YWL5534 | *MATα NUM1-yEGFP::spHIS5 bni1∆::TRP1 HIS3p:mRuby2-TUB1+3'UTR::URA3 kar9∆::HPH^R^ ura3-52 lys2-801 leu2-∆1 his3-∆200 trp1-∆63* | This study |
| YWL5609 | *MATa NUM1-yEGFP-CAAX::spHIS5 scs2∆::TRP1 scs22∆::KAN^R^ DYN1-3mCherry::HIS3 CFP-TUB1::URA3 ura3-52 lys2-801 leu2-∆1 his3-∆200 trp1-∆63* | This study |
| YWL5610 | *MATa NUM1-yEGFP-CAAX::spHIS5 DYN1-3mCherry::HIS3 CFP-TUB1::URA3 ura3-52 lys2-801 leu2-∆1 his3-∆200 trp1-∆63* | This study |
| YWL5611 | *MATα NUM1-yEGFP-CAAX::spHIS5 scs2∆::TRP1 scs22∆::KAN^R^ JNM1-3mCherry::HIS3 CFP-TUB1::URA3 ura3-52 lys2-801 leu2-∆1 his3-∆200 trp1-∆63* | This study |
| YWL5612 | *MATa NUM1-yEGFP-CAAX::spHIS5 JNM1-3mCherry::HIS3 CFP-TUB1::URA3 ura3-52 lys2-801 leu2-∆1 his3-∆200 trp1-∆63* | This study |
| YWL5649 | *MATa NUM1-yEGFP::spHIS5 scs2∆::TRP1 scs22∆::KAN nip100∆aa(2-103)-13myc::HPH^R^ ura3-52 lys2-801 leu2-∆1 his3-∆200 trp1-∆63* | This study |
| YWL5650 | *MATα NUM1-yEGFP::spHIS5 nip100∆aa(2-103)-13myc::HPH^R^ ura3-52 lys2-801 leu2-∆1 his3-∆200 trp1-∆63* | This study |
| YWL504 | *MATα dyn1∆::TRP1 ura3-52 lys2-801 leu2-∆1 his3-∆200 trp1-∆63* | This study |
